# Goodness-of-fit testing for meta-analysis of rare binary events

**DOI:** 10.1038/s41598-023-44638-x

**Published:** 2023-10-18

**Authors:** Ming Zhang, Olivia Y. Xiao, Johan Lim, Xinlei Wang

**Affiliations:** 1 Department of Statistics and Data Science, Southern Methodist University, Dallas, Texas, 75205 USA; 2 Highland Park High School, Dallas, Texas, 75205 USA; 3 Department of Statistics, Seoul National University, Seoul, 08826 Korea; 4 Department of Mathematics, University of Texas at Arlington, Arlington, Texas, 76019 USA; 5 Center for Data Science Research and Education, College of Science, University of Texas at Arlington, Arlington, Texas, 76019 USA

**Keywords:** Statistics, Medical research

## Abstract

Random-effects (RE) meta-analysis is a crucial approach for combining results from multiple independent studies that exhibit heterogeneity. Recently, two frequentist goodness-of-fit (GOF) tests were proposed to assess the fit of RE model. However, they tend to perform poorly when assessing rare binary events. Under a general binomial-normal framework, we propose a novel GOF test for the meta-analysis of rare events. Our method is based on pivotal quantities that play an important role in Bayesian model assessment. It further adopts the Cauchy combination idea proposed in a 2019 JASA paper, to combine dependent p-values computed using posterior samples from Markov Chain Monte Carlo. The advantages of our method include clear conception and interpretation, incorporation of all data including double zeros without the need for artificial correction, well-controlled Type I error, and generally improved ability in detecting model misfits compared to previous GOF methods. We illustrate the proposed method via simulation and three real data applications.

## Introduction

Meta-analysis is a valuable technique used in various fields, including medicine, biology, social sciences, and ecology, to combine information from multiple studies to increase inference reliability. A random-effects model (REM) is a popular choice in a meta-analysis, which assumes that the actual effect sizes of component studies $\theta _{i}$s follow a normal distribution with an overall mean $\theta _{0}$ and variance $\tau ^{2}$ (often referred to as the heterogeneity parameter). When $\tau ^{2}=0$, a REM is reduced to a fixed-effect model (FEM) with $\theta _{i}\equiv \theta _{0}$. REMs are preferred over FEMs in most scenarios because they account for the heterogeneity among studies and are, therefore, applicable to a broader range of scenarios^1^.

Among REMs, a generic model widely employed for binary and continuous outcomes uses the normal-normal hierarchical structure. For study *i*, let $y_{i}$ be the observed effect size (for binary data, $y_{i}$ is typically the log odds ratio), and $\sigma _{i}^{2}$ denotes the within-study variance (i.e., the sampling variation in study *i*). The generic model specifies $y_{i}|\theta _{i},\sigma _{i}^{2}\sim \text {N}\left( \theta _{i},\sigma _{i}^{2}\right)$ and $\theta _{i}\sim \text {N}\left( \theta _{0},\tau ^{2}\right)$. However, for rare binary outcomes, the normal approximation for $y_{i}$ given $\theta _{i}$ and $\sigma _{i}^{2}$ may not work well due to the sparsity or small sample sizes. Alternatively, the binomial-normal (BN) hierarchal structure is a popular substitute for the normal approximation. It assumes that the number of observed events in the treatment (control) group for study *i*, denoted by $x_{i2}\left( x_{i1}\right)$, follows a binomial distribution with the total number of subjects $n_{i2}\left( n_{i1}\right)$ and event probability $p_{i2}\left( p_{i2}\right)$. The logit transformed probabilities are then assumed to be distributed normally in the second hierarchy, where the log odds scale measures the effect size $\theta _{i}$. Several variations of the BN framework have been proposed. Bhaumik et al.^2^ assumed $\text {logit}\left( p_{i1}\right) =\mu _{i}$ and $\text {logit}\left( p_{i2}\right) =\mu _{i}+\theta _{i}$, where $\mu _{i}\sim \text {N}(\mu _{0},\sigma ^{2})$ denotes logit-transformed background incidence rate for study *i*. Smith, Spiegelhalter, and Thomas^3^ considered the equal variance between the control and treatment group by defining $\text {logit}\left( p_{i1}\right) =\mu _{i}-\theta _{i}/2$ and $\text {logit}\left( p_{i2}\right) =\mu _{i}+\theta _{i}/2$. Li and Wang^4^ proposed a more flexible model by defining $\text {logit}\left( p_{i1}\right) =\mu _{i}-\omega \theta _{i}$ and $\text {logit}\left( p_{i2}\right) =\mu _{i}+(1-\omega )\theta _{i}$, where the new parameter $\omega$ adjusts the variance ratio between two arms and the previous two models can be viewed as special cases by assigning $\omega =0$ and 1/2, respectively. To avoid the assumption of independency between $\mu _{i}$ and $\theta _{i}$, Houweilingen, Zwinderman and Stijnen^5^ proposed the use of a bivariate normal distribution for modeling $(\text {logit}\left( p_{i1}\right) ,\text {logit}\left( p_{i2}\right) )$, which allows any correlation structure between $\text {logit}\left( p_{i1}\right)$ and $\text {logit}\left( p_{i2}\right)$ in order to test the effects of each variable.

All the models discussed above make a common assumption that the true effect sizes $\theta _{i}'s$ follow a normal distribution $\theta _{i}\sim \text {N}\left( \theta _{0},\tau ^{2}\right)$. While this assumption is convenient for mathematical purposes, it may not always hold in reality as the distribution of true effect sizes across different studies could have any shape. Therefore, conducting a goodness-of-fit (GOF) test is crucial before drawing conclusions or making inferences, since a misspecified model may yield misleading results^6, 7^. Researchers have come up with various solutions to test the normality of models. Recently, Chen, Zhang, and Li^8^ proposed a parametric bootstrap-type GOF test, mainly focused on the generic REM. Subsequently, Wang and Lee^9^ developed a standardization framework to evaluate the normality assumption. It avoids the need to generate reference distributions and is therefore computationally efficient. However, their methods require continuity corrections when encountering single or double-zero studies, which can impact both Type I error rates and statistical power. Furthermore, those who previously proposed methods did not investigate their approaches numerically under different background incidence rates, especially when dealing with rare binary outcomes. This is an interesting and important aspect to explore in meta-analyses of binary outcomes.

In terms of Bayesian alternatives, no Bayesian approach has been considered for GOF testing in meta-analysis to our knowledge. We propose a novel GOF test for meta-analysis that utilizes the pivotal quantity (PQ) methodology proposed for Bayesian model assessment^10, 11^, and adapts the Cauchy combination test^12^, which combines dependent* p* values computed using posterior draws from Markov chain Monte Carlo (MCMC), to inform the final conclusion. A pivotal quantity is a function of data and model parameters whose distribution does not depend on unknown parameters. For instance, suppose $\theta =(\mu ,\sigma )\sim \pi$, and $\theta _{0}=(\mu _{0},\sigma _{0})$ is a random vector drawn from density $\pi$, which generates the normal data $\textbf{x}=\{x_{1},...,x_{n}\}$. Then $f(\textbf{x},\mu _{0},\sigma _{0})=\sum _{i=1}^{n}(\frac{x_{i}-\mu _{0}}{\sigma _{0}})^{2}\sim \chi _{n}^{2}$ is a pivotal quantity. Let $\tilde{\mu}$ and $\tilde{\sigma }$ be samples from the corresponding posterior distribution $p(\mu ,\sigma |\textbf{x})$. The PQ method is constructed based on the fact that $f(\textbf{x},\mu_{0},\sigma_{0})$ and $f(\textbf{x},\tilde{\mu},\tilde{\sigma})$ are identically distributed; that is, $f(\textbf{x},\tilde{\mu},\tilde{\sigma})\sim \chi _{n}^{2}$.

Our proposed method, called Improved Pivotal Quantities (IPQ), can detect a model failure at all levels in hierarchical models without extra computational cost. Additionally, it can be easily incorporated into standard Bayesian implementations and automatically accounts for all available data without requiring artificial corrections for rare binary events. While our method is suitable for general purposes, we focus primarily on its application for meta-analyses of rare binary outcomes.

The rest of this article is organized as follows. We first review Bayesian techniques that assess model adequacy, followed with a brief introduction to pivotal quantities and the Cauchy combination test. We then introduce our proposed method based on the generalized REM in Houweilingen, Zwinderman and Stijnen^5^ for meta-analysis of binary events. We also describe the Bayesian implementation of our method, including adapting the proposed IPQ method within the MCMC algorithm and considering different bivariate covariance priors. In the simulation section, we conduct simulation studies to evaluate our method’s performance in terms of Type I error rates and statistical power and compare it with other existing GOF methods. We also evaluate four different covariance priors based on estimating the overall treatment effect, the inter-study heterogeneity, and the correlation coefficient. In data examples section, we illustrate our method using three real data examples. The first example utilizes handedness and eye-dominance data from 54 studies, the second one employs Type 2 diabetes mellitus and gestational diabetes data from 20 studies, and the third uses GSTP1 gene and lung cancer data from 44 studies. We then end this paper with conclusions and discussions.

## Review of related bayesian work

**Figure 1:**
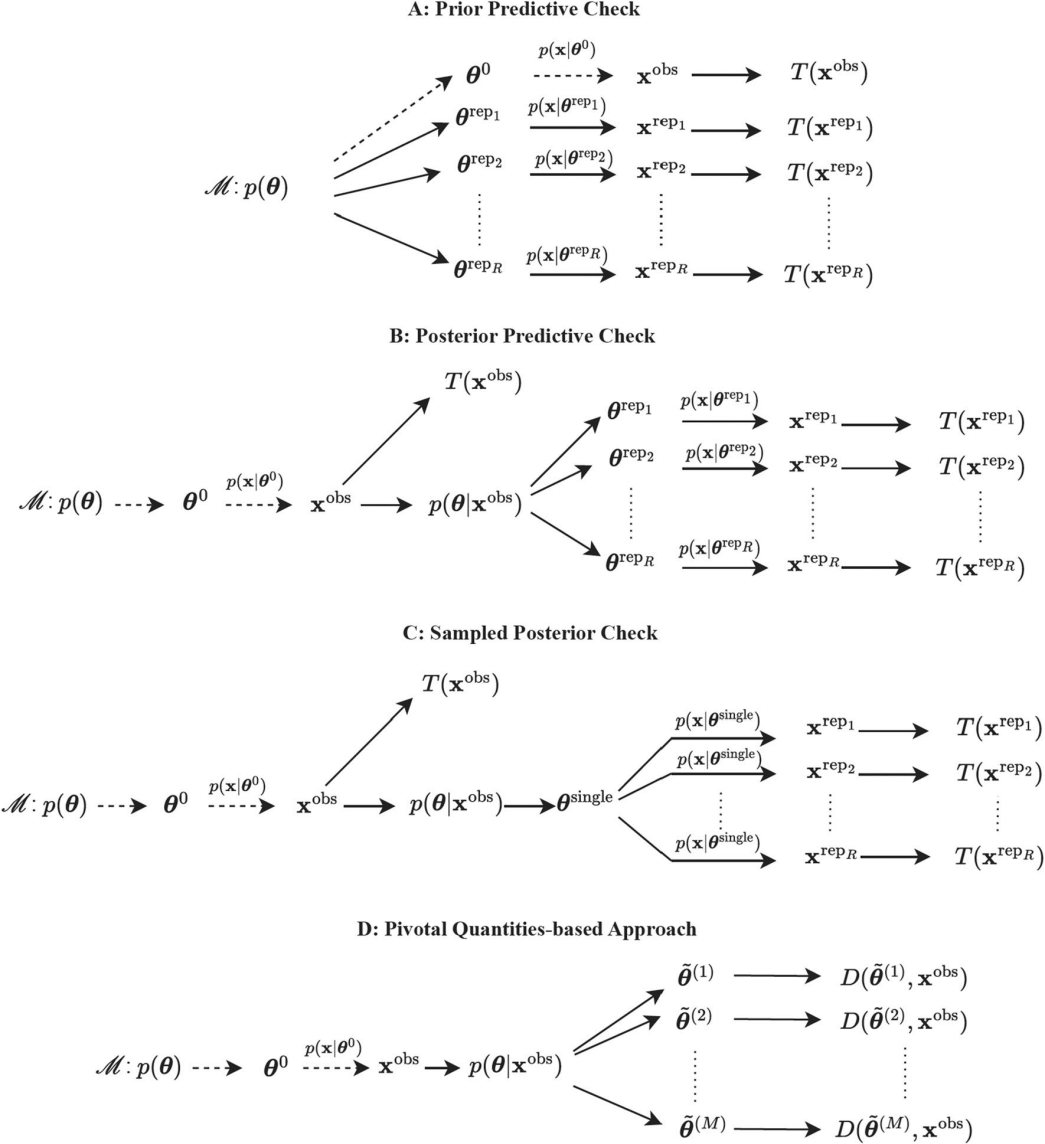
Schematic diagrams of different model diagnostic methods.

In current practice, Bayesian model diagnostics mainly fall into three categories: prior predictive, posterior predictive, and pivotal quantity-based approaches. See Figure 1 for illustration.

### Prior and posterior predictive checks

Suppose $\textbf{x}$ has a distribution function specified by $p\left( \textbf{x}|\varvec{\theta }\right)$, where $\varvec{\theta }$ represents the parameters of a model (say $\mathscr {M}$) under study. Let $\textbf{x}^{\text {obs}}$ denote the observed data and $\textbf{x}^{\text {rep}}$ denote the replicated data that are generated to mimic real data. Box^13^ recommended using the prior predictive distribution, $p\left( \textbf{x}\right) =\int p\left( \textbf{x}|\varvec{\theta }\right) p\left( \varvec{\theta }\right) d\varvec{\theta }$, as a reference distribution to generate $\textbf{x}^{\text {rep}}$ for comparing with $\textbf{x}^{\text {obs}}$. The steps to obtain the prior predictive distribution are illustrated in Figure 1A. Given $\varvec{\theta }^{\text {rep}_{j}}$ drawn from the prior $p\left( \varvec{\theta }\right)$ for $j=1,..,R$, we draw $\textbf{x}^{\text {rep}_{j}}$ from the sampling distribution $p\left( \textbf{x}|\varvec{\theta }^{\text {rep}_{j}}\right)$. We then use $T\left( \textbf{x},\varvec{\theta }\right)$ or $T\left( \textbf{x}\right)$, a function of data and model parameters or a function of data alone, to measure the discrepancy between data and model assumptions. Here, we take $T\left( \textbf{x}\right)$ as an example for simplicity, evaluated at both $\textbf{x}^{\text {obs}}$ and $\textbf{x}^{\text {rep}_{j}}$ for all *j*. The model misfit can be concluded if $T\left( \textbf{x}^{\text {obs}}\right)$ is unlikely from the reference distribution formed by $T\left( \textbf{x}^{\text {rep}_{j}}\right)$’s. However, prior predictive checks might be problematic when using improper or weakly-informative priors, which are commonly used in practice^14^.

Gelman, Meng, and Stern^15^ proposed model assessment using the posterior predictive distribution, defined as $p\left( \textbf{x}^{\text {rep}}|\textbf{x}^{\text {obs}}\right) =\int p\left( \textbf{x}^{\text {rep}}|\varvec{\theta }\right) p\left( \varvec{\theta }|\textbf{x}^{\text {obs}}\right) d\varvec{\theta }$. As shown in Figure 1B, replicated data $\textbf{x}^{\text {rep}_{j}}$ are generated using $\varvec{\theta }^{\text {rep}_{j}}$ from the posterior distribution $p\left( \varvec{\theta }|\textbf{x}^{\text {obs}}\right)$. Then, the reference distribution based on the chosen discrepancy function $T\left( \textbf{x}\right)$ can be computed. The Bayesian posterior predictive *p* value can be obtained as $P\left[ T\left( \textbf{x}^{\text {rep}}\right) \ge T\left( \textbf{x}^{\text {obs}}\right) |\textbf{x}^{\text {obs}}\right]$ to quantitatively detect the model misfit.

The posterior predictive check has gained increasing popularity in Bayesian model checking due to its straightforward implementation via Monte Carlo Markov Chain (MCMC) algorithms. However, there are two major limitations associated with this type of approach. Firstly, unlike the traditional *p* value, the posterior predictive *p* value does not follow a uniform distribution under the null hypothesis of no lack of fit, making it difficult to interpret and assess the level of evidence against the null hypothesis^16^. Secondly, the method has almost no power to detect failures from the second or deeper layers in hierarchical models^11, 17^.

To overcome the non-uniformity problem, a potential solution is to calibrate the posterior predictive *p* value so that the calibrated *p* value follows a uniform distribution asymptotically^18^. However, the statistical power of using the calibrated *p* value has not been investigated yet. To avoid this issue, Bayarri and Berger^19^ proposed two new types of *p* values: the conditional predictive *p* values and the partial posterior predictive *p* values. Bayarri and Castellanos^16^ further extended the partial posterior predictive method to test the second layer of hierarchical models, which avoids “using data twice.” However, as mentioned in Johnson^20^, the partial posterior strategy is typically not straightforward to implement beyond normal-family problems. More recently, Gosselin^21^ and Zhang^22^ recommended randomly drawing a single value $\varvec{\theta }^{\text {single}}$ from the posterior distribution $\pi \left( \varvec{\theta }|\textbf{x}^{\text {obs}}\right)$ to generate $\textbf{x}^{\text {rep}}$, namely sampled posterior check (Figure 1C). The corresponding *p* values is distributed uniformly when the data model is correctly specified, and the approach achieved higher power than the original posterior predictive check for detecting model misfit^21^.

### Pivotal quantity methodology

Johnson^10^ pioneered the use of pivotal quantities (PQ) to detect model misfit, and Yuan and Johnson^11^ extended upon the methodology so that it can be applied to any level of hierarchical models. Since it does not involve replicated data, there is no need to distinguish $\textbf{x}^{\text {obs}}$ and $\textbf{x}^{\text {rep}}$, and $\textbf{x}$ is directly used for observed data.

A pivotal quantity, denoted by $D\left( \textbf{x};\varvec{\theta }\right)$, is a function of both data $\textbf{x}$ and model parameters $\varvec{\theta }$. It possesses a sampling distribution *F* that is both known and invariant when evaluated at $\varvec{\theta }^{0}$, the “true” (data-generating) value of $\varvec{\theta }$; that is, $D\left( \textbf{x};\varvec{\theta }^{0}\right) \sim F$. Johnson^10^ shows that $D\left( \textbf{x};\tilde{\varvec{\theta }}\right)$ and $D\left( \textbf{x};\varvec{\theta }^{0}\right)$ are identically distributed, where $\tilde{\varvec{\theta }}$ is drawn from the posterior distribution $p\left( \varvec{\theta }|\textbf{x}\right)$. Based on this result, the approach to model assessment involves two main steps^10^. The first is to select a pivotal discrepancy measure $D\left( \textbf{x};\varvec{\theta }\right)$ with a known reference distribution *F*, and, the second step is to evaluate the model fit by determining whether $D\left( \textbf{x};\tilde{\varvec{\theta }}\right)$ can be considered as a draw from *F*. However, when conducting a GOF test for the second or deeper layers in hierarchical models, one may encounter difficulties since $D\left( \textbf{x};\varvec{\theta }\right)$ depends on $\textbf{x}$, but these layers usually involve no data. For this reason, Yuan and Johnson^11^ extended the method by defining the pivotal quantity *D* as a function of model parameters only and further showed that $D\left( \varvec{\theta }^{0}\right)$ and $D\left( \tilde{\varvec{\theta }}\right)$ have identical distributions. This allows the application of pivotal quantities and the corresponding reference distributions to diagnose model inadequacy at any level of a hierarchical model.

As shown in Figure 1D, after drawing $\tilde{\varvec{\theta }}^{(i)}$ from $p\left( \varvec{\theta }|\textbf{x}\right)$, $D\left( \textbf{x};\varvec{\theta }\right)$ is evaluated at $\tilde{\varvec{\theta }}^{(i)}$ for $i=1,...,M$. Then, each $D\left( \textbf{x},\tilde{\varvec{\theta }}^{(i)}\right)$ has the same distribution as $D\left( \textbf{x},\varvec{\theta }^{\text {0}}\right)$. For example, suppose $D\left( \textbf{x},\varvec{\theta }^{\text {0}}\right) \sim \text {N}(0,1)$, then under the null hypothesis of no lack of fit, $D\left( \textbf{x},\tilde{\varvec{\theta }}^{(i)}\right) \sim \text {N}(0,1)$ for each *i* marginally. To test normality, Johnson^10^ suggested using a formal approach such as the Shapiro-Wilks test, and different p values from the tests on $\left( \textbf{x},\tilde{\varvec{\theta }}^{(i)}\right)$ are calculated for $i=1,...M.$ However, combining those p values is not as straightforward as using Fisher’s combination test. This is because the p values are derived from posterior samples using the same dataset and so are dependent with an unknown covariance structure.

To address this issue, Johnson^10^ suggested that one could avoid generating multiple draws from the same dataset by utilizing the prior-predictive distribution from Dey et al.^17^, which suggested generating 1000 replicated datasets, $\textbf{x}^{\text {rep}_i}$ for $i=1,...,1000$, from $p\left( \textbf{x}\right)$ as illustrated in Figure 1A. For each replicated dataset $\textbf{x}^{\text {rep}_i}$, Bayesian data analysis is performed to obtain the corresponding posterior distribution $p(\varvec{\theta }|\textbf{x}^{\text {rep}_i})$, then a single $\tilde{\varvec{\theta }}^{\text {rep}_i}$ is randomly sampled from the posterior, and one *p* values from testing the normality using the pivotal quantity is computed. This results in 1000 independent *p* values. Standard approaches, such as Fisher’s test, can then be employed to draw a conclusion. However, this method may suffer from two limitations. Firstly, using 1000 replicated datasets can be computationally intensive since the same MCMC procedure needs to run 1000 times to draw independent posterior samples. Secondly, non-informative priors may not necessarily generate reasonable datasets. Considering these difficulties, Johnson^10^ recommended finding probabilistic bounds on dependent *p* values using the properties of order statistics derived from Gascuel and Caraux^23^ and Rychlik^24^.

Let $x_{(1)},..,x_{(M)}$ denote order statistics from a dependent sample of random variables, where each has distribution function *F*, and let $F_{k:M}$ denote the distribution function for the k-th order statistic out of *M*. Then, the bound of $F_{k:M}$ can be written as

1 $$\begin{aligned} F_{k:M}\left( t\right) \ge \max \left\{ 0,\frac{MF\left( t\right) -k+1}{M-k+1}\right\} . \end{aligned}$$

Let $p_{1},...,p_{M}$ be dependent *p* values for $m=1,...,M$. Under the null, each *p* values should be distributed uniformly on $\left( 0,1\right)$, implying $F\left( t\right) =t$ in Eq. (1). Let $x_{i}=-p_{i}$, Li, Wu and Feng^25^ showed that the Eq. (1) becomes

$$\begin{aligned} F_{k:M}\left( t\right) \le \min \left( 1,t\frac{M}{k}\right) , \end{aligned}$$

which means that a *p* value upper bound for the observed k-th order statistic $p_{(k)}^{\text {obs}}$ is $\min \left( 1,p_{(k)}^{\text {obs}}\frac{M}{k}\right) .$ To avoid choosing the value of *k*, they suggested reporting the minimum upper bound such that $p_{\text {min}}=\text {min}\left\{ \min \left( 1,p_{(k)}^{\text {obs}}\frac{M}{k}\right) \right\} _{k=1,...,M}.$ Yuan and Johnson^11^ advocated using the rule-of-thumb value of 0.25 as a cutoff for declaring the model misfit in practice; that is, reject the null hypothesis $\mathscr{H}_{0}$ if $p_{\text {min}}<0.25$. However, the proposal may be liberal, and our simulation studies in the simulation section show that 0.25 is not necessarily a good choice and it is hard to select an optimal cutoff to balance the trade-off between Type I error and power.

## Method

### The generalized REM for meta-analysis of binary events

Suppose a meta-analysis contains *I* independent studies, and for the $i^{th}$ study, let $x_{i1}\left( x_{i2}\right)$ be the number of observed events in the control (treatment) group, which follows a binomial distribution with the total number of subjects $n_{i1}\left( n_{i2}\right)$ and corresponding event probability $p_{i1}\left( p_{i2}\right)$. Let $\phi _{i1}\left( \phi _{i2}\right)$ denote the logit-transformed $p_{i1}\left( p_{i2}\right)$, i.e., $\phi _{ij}\equiv \ln \left( \frac{p_{ij}}{1-p_{ij}}\right)$. Then the generalized binomial-normal REM in Houweilingen, Zwinderman and Stijnen^5^ can be written as

2 $$\begin{aligned} \begin{array}{cc} x_{i1}\thicksim \text {Bin}\left( n_{i1},p_{i1}\right) ,\,x_{i2}\thicksim \text {Bin}\left( n_{i2},p_{i2}\right) ,\\ \text {logit}\left( p_{i1}\right) =\phi _{i1},\,\text {logit}\left( p_{i2}\right) =\phi _{i2},\\ \left( \begin{array}{c} \phi _{i1}\\ \phi _{i2} \end{array}\right) \sim \text {N}\left( \left( \begin{array}{c} \mu _{1}\\ \mu _{2} \end{array}\right) ,\left( \begin{array}{cc} \sigma _{1}^{2} &{} \rho \sigma _{1}\sigma _{2}\\ \rho \sigma _{1}\sigma _{2} &{} \sigma _{2}^{2} \end{array}\right) \right) , \end{array} \end{aligned}$$

where $(\phi _{i1},\phi _{i2})$ is modeled by a bivariate normal distribution with an arbitrary covariance structure. We further define the treatment effect $\theta _{i}=\phi _{i2}-\phi _{i1}$ for study *i*, which follows a univariate normal distribution with an overall mean effect $\theta _{0}=\mu _{2}-\mu _{1}$ and the heterogeneity $\tau ^{2}=\sigma _{1}^{2}+\sigma _{2}^{2}-2\rho \sigma _{1}\sigma _{2}$.

The generalized REM builds a strong connection to many well-established models^26^. For example, the model in Li and Wang^4^ and Zhang et al.^27^ is a special case of model (2), yielding

$$\begin{aligned} \left( \begin{array}{c} \phi _{i1}\\ \phi _{i2} \end{array}\right) \sim \text {N}\left( \left( \begin{array}{c} \mu -\omega \theta \\ \mu +\left( 1-\omega \right) \theta \end{array}\right) ,\left( \begin{array}{cc} \omega ^{2}\tau ^{2}+\sigma ^{2} &{} \sigma ^{2}-\omega \left( 1-\omega \right) \tau ^{2}\\ \sigma ^{2}-\omega \left( 1-\omega \right) \tau ^{2} &{} \left( 1-\omega \right) ^{2}\tau ^{2}+\sigma ^{2} \end{array}\right) \right) , \end{aligned}$$

where in (2), $\sigma _{1}^{2}=\omega ^{2}\tau ^{2}+\sigma ^{2}$, $\sigma _{2}^{2}=\left( 1-\omega \right) ^{2}\tau ^{2}+\sigma ^{2}$, and $\rho =\frac{\sigma ^{2}-\omega \left( 1-\omega \right) \tau ^{2}}{\sqrt{\left( \omega ^{2}\tau ^{2}+\sigma ^{2}\right) \left( \left( 1-\omega \right) ^{2}\tau ^{2}+\sigma ^{2}\right) }}$. As mentioned in the introduction, we can let $\omega$ be 0 or 0.5, which further reduces the model to the one in Bhaumik et al.^2^ or Smith, Spiegelhalter, and Thomas^3^, respectively. Thus, model (2) is regarded as the most generalized binomial-normal model with fewer assumptions, so we choose it as the basis to design the GOF test for detecting non-normality of $\theta _{i}$’s.

Let $\varvec{\Theta }=\left\{ \varvec{\phi }_{1},\varvec{\phi }_{2},\mu _{1},\mu _{2},\varvec{\Sigma }\right\}$ be all parameters in (2), where $\varvec{\phi }_{j}=\left\{ \phi _{1j},...,\phi _{Ij}\right\}$ for $j=1,2$ and $$\varvec{\Sigma }=\left( \begin{array}{cc} \sigma _{1}^{2} &{} \rho \sigma _{1}\sigma _{2}\\ \rho \sigma _{1}\sigma _{2} &{} \sigma _{2}^{2} \end{array}\right) .$$ Let $\textbf{X}=\left\{ x_{i1},x_{i2}\right\} _{i=1}^{I}$ be the data. Then the full probability model is given by

$$\begin{aligned} p\left( \textbf{X},\varvec{\Theta }\right) =\prod _{i=1}^{I}p(x_{i1},x_{i2}|\phi _{i1},\phi _{i2})p(\phi _{i1},\phi _{i2}|\mu _{1},\mu _{2},\varvec{\Sigma })p(\mu _{1},\mu _{2},\varvec{\Sigma }), \end{aligned}$$

where

$$\begin{aligned} p(x_{i1},x_{i2}|\phi _{i1},\phi _{i2})=\left( \begin{array}{c} n_{i1}\\ x_{i1} \end{array}\right) \left( \begin{array}{c} n_{i2}\\ x_{i2} \end{array}\right) \frac{(e^{\phi _{i1}})^{x_{i1}}}{(1+e^{\phi _{i1}})^{n_{i1}}}\frac{(e^{\phi _{i2}})^{x_{i2}}}{(1+e^{\phi _{i2}})^{n_{i2}}}, \end{aligned}$$

$p(\phi _{i1},\phi _{i2}|\mu _{1},\mu _{2},\varvec{\Sigma })$ is the density function of the bivariate normal distribution; $p(\mu _{1},\mu _{2},\varvec{\Sigma })$ is the joint prior distribution on hyper-parameters introduced by the bivariate normal distribution of $(\phi _{i1},\phi _{i2})$.

### The proposed GOF test

Inspired by previous research, we propose a novel GOF test and demonstrate its applicability in the context of meta-analysis of (rare) binary events. Our approach involves defining the null hypothesis, denoted by $\mathscr {H}_{0}$, which assumes normality for the true effect sizes $\theta _{i}$s, a prevailing assumption made in meta-analysis. The alternative hypothesis, denoted by $\mathscr{H}_{1}$, is formulated as any departure from $\mathscr {H}_{0}$. In other words, we aim to detect this specific departure from the bivariate normal model assumed for the second layer of the generalized REM, where we can draw our conclusion about the presence of an overall treatment effect $\theta _{0}$ and between-study heterogeneity $\tau ^{2}$.

Let $\varvec{\Theta }_{i}^{*}=\left( \phi _{i1}^{*},\phi _{i2}^{*},\mu _{1}^{*},\mu _{2}^{*},\sigma _{1}^{2*},\sigma _{2}^{2*},\rho ^{*}\right)$ be data-generating parameter values for study *i*, and $\tilde{\varvec{\Theta }}_{i}^{(m)}$ be the corresponding $m^{th}$ draw from the joint posterior distribution $p\left( \varvec{\Theta }|\textbf{X}\right)$ for $m=1,...,M$. We define a discrepancy measure to capture the deviation from $\mathscr {H}_{0}$, namely

$$\begin{aligned} D\left( \varvec{\Theta }_{i}^{*}\right) =\frac{\phi _{i1}^{*}-\phi _{i2}^{*}-\theta _{0}^{*}}{\tau ^{*}}=\frac{\phi _{i1}^{*}-\phi _{i2}^{*}-\left( \mu _{1}^{*}-\mu _{2}^{*}\right) }{\sqrt{\sigma _{1}^{2*}+\sigma _{2}^{2*}-2\rho ^{*}\sigma _{1}^{*}\sigma _{2}^{*}}}, \end{aligned}$$

which is a pivotal quantity and follows a standard normal distribution under $\mathscr {H}_{0}$. Furthermore, as pointed out by one of our reviewers, this measure can be viewed as the study-specific effect in units of standard deviations, for each study and each posterior draw. Then, according to Yuan and Johnson^11^,

3 $$\begin{aligned} D\left( \tilde{\varvec{\Theta }_{i}}^{(m)}\right) =\frac{\tilde{\phi }_{i1}^{(m)}-\tilde{\phi }_{i2}^{(m)}-\tilde{\theta }_{0}^{(m)}}{\tilde{\tau }^{(m)}}=\frac{\tilde{\phi }_{i1}^{(m)}-\tilde{\phi }_{i2}^{(m)}-\left( \tilde{\mu }_{2}^{(m)}-\tilde{\mu }_{2}^{(m)}\right) }{\sqrt{\tilde{\sigma }_{1}^{2(m)}+\tilde{\sigma }_{2}^{2(m)}-2\tilde{\rho }^{(m)}\tilde{\sigma }_{1}^{(m)}\tilde{\sigma }_{2}^{(m)}}} \end{aligned}$$

has a distribution identical to $D\left( \varvec{\Theta }_{i}^{*}\right)$; that is, $D\left( \tilde{\varvec{\Theta }_{i}}^{(m)}\right) \sim \text {N}\left( 0,1\right)$ for every *m* marginally.

Conducting a standard normality test using the pivotal quantities in (3) based on a single draw is straightforward, but this sampled posterior approach can be problematic since the vagaries of randomness can produce a sample that seems unwise. Alternatively, combining multiple MCMC draws to draw a conclusion was recommended in Johnson^10^ and Yuan and Johnson^11^, where probabilistic bounds of the order statistics of the *p* values are used to combine the dependent *p* values. Here, we propose to use the Cauchy combination idea^12^ to combine the dependent *p* values.

Consider $p_{i}$ as the *p* values obtained from the i-th statistical test, and $\omega _{i}$ as the corresponding nonnegative weight that sums up to 1. Liu and Xie^12^ introduced the Cauchy combination test and demonstrated that, subject to certain regularity conditions, the tail of a test statistic that linearly combines individual transformed *p* values can be well approximated by a standard Cauchy distribution under the null hypothesis. Specifically, if there are *k* *p* values, then the test statistic is given by $T=\sum _{i=1}^{k}\omega _{i}\tan \left\{ \left( 0.5-p_{i}\right) \pi \right\}$, where the weight $\omega _{i}$ is typically set to 1/*k* in the absence of any prior information. The Cauchy combination test has several salient features. Firstly, the test, by leveraging the Cauchy distribution, the test has a simple analytical formula to compute the *p* value. Next, unlike classical Fisher’s test^28^ or other common tests for combining *p* values, such as the minimum *p* value test^29^, the Berk-Jones test^30^ and the higher criticism test^31^, the Cauchy combination test handles *p* values from correlated statistical tests and remains valid for arbitrary correlation structures. Finally, the test works well even if one main assumption required for the test, the bivariate normality between any two test statistics generating the *p* values, is not satisfied. Thus, $p_{i}$s can be from non-normal typed tests (i.e., those with test statistics that are not normally distributed), such as the Shapiro-Wilk test^32^, the Cramer-von Mises test^33–35^ and the Anderson-Darling test^35, 36^.

In summary, the proposed GOF test, namely Improved Pivotal Quantities (IPQ), can be outlined by the following steps:

**Step 1:** Given *I* independent studies, randomly sample $\tilde{\varvec{\Theta }}_{i}^{(m)}$ from the joint posterior distribution $p\left( \varvec{\Theta }|\textbf{X}\right)$ via MCMC for $i=1,...,I$ and $m=1,...M$.

**Step 2:** Calculate $D\left( \tilde{\varvec{\Theta }_{i}}^{(m)}\right)$ in Eq. (3) for all *i* and *m*. For each $m^{th}$ draw, use $\left\{ D\left( \tilde{\varvec{\Theta }_{i}}^{(m)}\right) \right\} _{i=1}^{I}$ to conduct a formal normality test (e.g. Shapiro-Wilk test) to get its *p* values, say $p^{(m)}.$

**Step 3:** Compute the test statistic $T_{0}=\sum _{m=1}^{M}\frac{\tan \left\{ \left( 0.5-p^{(m)}\right) \pi \right\} }{M}$, and calculate the corresponding *p* values using the formula $p^{*}=\frac{1}{2}-\frac{\text {arctan}T_{0}}{\pi }.$ Then, we will reject the $\mathscr {H}_{0}$ if $p^{*}<\alpha$ with a pre-specified significance level (e.g., $\alpha =0.01,0.05,0.1$).

### Bayesian implementation with different covariance priors

We now pivot the discussion to prior specification and the Bayesian implementation. We use a Hamiltonian Monte Carlo (HMC) algorithm via Stan (version 2.19.1)^37^ in conjunction with R^38^ to fit models with different priors discussed below. For each dataset, we run the algorithm with 5000 burn-in iterations and 5000 additional sampling iterations. The convergence of MCMC chains is detected using the Gelman-Rubin diagnostic^39^.

We start with the prior choices for logit-transformed mean effects $\mu _{1}$ and $\mu _{2}$ for the control and treatment groups, where we consider diffuse uniform priors such that $\mu _{j}\sim \text {U}\left( \text {L}_{\mu _{j}},\text {U}_{\mu _{j}}\right)$ for $j=1,2$. To define the range, we get rough estimates $\hat{\mu }_{ij}$ for all *I* studies, $\hat{\mu }_{ij}=\ln \frac{x_{ij}+0.5}{n_{ij}-x_{ij}+0.5}$. Then, we define the lower bound $\text {L}_{\mu _{j}}=\min _{i,j}\left\{ \hat{\mu }_{ij}\right\} -c$ and upper bound $\text {U}_{\mu _{j}}=\max _{i,j}\left\{ \hat{\mu }_{ij}\right\} +c$, where we let $c=5$ as in Bai et al.^40^ so that the priors are conservative enough to contain all plausible values.

Regarding the prior for the covariance matrix $\varvec{\Sigma }$, several commonly used conjugate priors are available, including the independent prior (IND) that assumes mutual independence *a priori* among the elements of $\varvec{\Sigma }$^41^, the inverse Wishart prior (IW)^42^ and the hierarchical inverse Wishart prior (HIW)^43^. Other alternatives include the scaled inverse Wishart prior (SIW)^44^ and the prior based on the separation strategy (SS)^45^. The Bayesian inference of a covariance matrix is highly sensitive to different choices of priors, and several studies have compared the performance of various priors. For example, Alvarez, Niemi and Simpson^46^ compared four different priors (IW, HIW, SIW and SS) in the multivariate normal model and found that the IW prior performed the worst among all the four, especially when the true variances were small. Rúa, Mazumdar and Strawderman^41^ conducted extensive simulation by comparing 38 priors, including IW, HIW, and IND, with different hyper-parameter specifications in multivariate Bayesian meta-analysis models. They found that the IW prior had overall poor performance, while the HIW prior had much more consistent performance across all scenarios examined. Akinc and Vandebroek^47^ focused on the same priors used in Alvarez, Niemi and Simpson^46^ and investigated Bayesian inference of the covariance matrix in mixed logit models. They suggested using different priors to check the robustness of the results but recommended avoiding the IW prior. To the best of our knowledge, the impact of different covariance priors on BN models in the context of meta-analysis of rare binary events has not been investigated. Thus, we aim to address the gap and access how these priors perform under rare binary settings. Below we briefly review four classes of priors, including IW, HIW, SS , and SIW, and their performance will be assessed in the simulation section.

#### Inverse Wishart prior

Due to the conjugacy property, the IW prior is often used as a default choice for covariance matrices. The density function of the IW prior $\text {IW}\left( \nu ,\textbf{H}\right)$ is defined as $p\left( \varvec{\Sigma }\right) \propto \left| \varvec{\Sigma }\right| ^{-\frac{\left( \nu +3\right) }{2}}\exp \left\{ -\frac{1}{2}\text {trace}\left( \textbf{H}\varvec{\Sigma }^{-1}\right) \right\}$, where $\nu >0$ is the number of degrees of freedom and $\textbf{H}$ is a symmetric scale matrix with two dimensions. The marginal distribution of the correlation parameter $\rho$ in $\varvec{\Sigma }$ is $p\left( \rho \right) \propto \left( 1-\rho ^{2}\right) ^{\frac{\left( \nu -3\right) }{2}}$ when $\textbf{H}$ is a diagonal matrix. If $\nu =3$, $\rho$ follows $\text {U}\left( -1,1\right)$^45^. For our model, the conditional posterior distribution of $\varvec{\Sigma }$ is given by $p\left( \varvec{\Sigma }|\varvec{\phi }_{1},\varvec{\phi }_{2},\mu _{1},\mu _{2}, {\textbf{x}}\right) \sim \text {IW}\left( \nu +I,\textbf{H}+\varvec{\Lambda }_{\mu }\right)$, where

$$\begin{aligned} \begin{array}{cc} \varvec{\Lambda }_{\mu }=\left( \begin{array}{cc} \sum _{i=1}^{I}\left( \phi _{i1}-\mu _{1}\right) ^{2} &{} \sum _{i=1}^{I}\left( \phi _{i1}-\mu _{1}\right) \left( \phi _{i2}-\mu _{2}\right) \\ \sum _{i=1}^{I}\left( \phi _{i1}-\mu _{1}\right) \left( \phi _{i2}-\mu _{2}\right) &{} \sum _{i=1}^{I}\left( \phi _{i2}-\mu _{2}\right) ^{2} \end{array}\right) .\end{array} \end{aligned}$$

While the IW prior is a popular choice in Bayesian analysis due to its mathematical convenience, it also has limitations. One issue is that selecting the appropriate degrees of freedom $\nu$ and scaled matrix $\textbf{H}$ can be challenging. Although these are often set to default values of 3 and an identity matrix, respectively, recent studies by Rúa et al.^41^ and Akinc and Vandebroek^47^ have shown that these choices may not always be suitable. Another limitation is that the IW prior implies a strong relationship between variance and correlation, which can bias inference. Specifically, smaller variances are associated with correlation coefficients $\rho$ around 0, while larger variances correspond to $\rho$ approaching −1 or 1. This dependency can be problematic when interpreting results and drawing conclusions from statistical analyses.

#### Hierarchical inverse Wishart prior

Huang and Wand^43^ proposed a two-layer hierarchical prior that builds upon the work of Wand et al.^48^ and Armagan, Artin et al.^49^, who showed that a half-t distribution can be expressed as a scale mixture of an inverse gamma distribution. In our case, the dimension of $\varvec{\Sigma }$ is two, so that their hierarchical prior is defined as $p\left( \varvec{\Sigma }|a_{1},a_{2}\right) \sim \text {IW}\left( \nu +1,\mathbf {\textbf{H}}^{*}\right)$, where $\mathbf {\textbf{H}}^{*}=2\nu \text {diag}\left( 1/a_{1},1/a_{2}\right)$, $a_{j}\sim \text {Inverse-Gamma}\left( 1/2,1/A_{j}^{2}\right)$ for $j=1,2$, $\nu >0$, and $A_{j}>0$ is typically assigned a large value (e.g. $10^{5}$) to indicate non-informativeness. They also showed that the marginal distribution of the correlation coefficient $\rho$ is uniform on $\left( -1,1\right)$ for bivariate cases when $\nu =2$. Compared to the IW prior, the HIW prior provides increased flexibility in the choice of the scaled matrix while retaining the conjugacy properties. In our model, the conditional posterior distribution of $\varvec{\Sigma }$ and $a_{j}$ for $j=1,2$ now become

$$\begin{aligned} \begin{array}{cc} p\left( \varvec{\Sigma }|\varvec{\phi }_{1},\varvec{\phi }_{2},\mu _{1},\mu _{2},a_{1},a_{2}, { {\textbf{x}}}\right) \propto \text {IW}\left( \nu +I+1,\varvec{\Lambda }_{\mu }+ {\textbf{H}}^{*}\right) ,\\ p\left( a_{j}|\varvec{\Sigma }, {\textbf{x}}\right) {\mathop {\sim }\limits ^{\text {ind}}}\text {Inverse-Gamma}\left( \frac{\nu }{2}+1,\nu \left( \Sigma ^{-1}\right) _{jj}+A_{j}^{-2}\right) , \end{array} \end{aligned}$$

where $\left( \varvec{\Sigma }^{-1}\right) _{jj}$ denotes the $\left( j,j\right)$ entry of $\varvec{\Sigma }^{-1}$, and we set $\nu =2$. However, Alvarez, Niemi and Simpson^46^ pointed out that, compared to the IW prior, the HIW prior is capable of reducing, but not eliminating, the dependency between variance and correlation.

#### Separation strategy

Barnard, McCulloch and Meng^45^ introduced a prior class known as the separation strategy (SS) that decomposes a covariance matrix $\varvec{\Sigma }$ into a diagonal matrix $\mathbf {\textbf{S}}$ of standard deviations (SDs) and a correlation matrix $\mathbf {\textbf{R}}$, resulting in $\varvec{\Sigma }=\mathbf {\textbf{SRS}}$. Specifically, for bivariate data, $\mathbf {\textbf{S}}=\text {diag}\left( \sigma _{1},\sigma _{2}\right)$ and $$\mathbf {\textbf{R}}=\left( \begin{array}{cc} \rho &{} 1\\ 1 &{} \rho \end{array}\right)$$. The SS prior assigns independent priors for the SDs and correlations, which eliminates the association between variance and correlation, setting it apart from the IW and HIW priors. Posterior computation with the SS prior is usually done via the Hamiltonian Monte Carlo (HMC) algorithm^50^, which was later improved by the No-U-Turn sampler^51^ in Stan. In the Stan manual^37^, the recommended hyperprior settings for the SS prior are $\sigma _{j}\sim \text {Cauchy}\left( 0,2.5\right)$ constrained by $\sigma _{j}>0$ for $j=1,2$ and $\mathbf {\textbf{R}}\sim \text {LKJCorr}\left( 1\right)$, where $\text {LKJCorr}\left( 1\right)$ denotes the $\text {LKJ}$ prior from Lewandowski et al.^52^ with a shape parameter of 1. However, implementing the specific SS prior still requires intensive posterior computation. On the other hand, the IND prior is the simplest among the SS class, which assigns independent priors on $\left( \sigma _{1}^{2},\sigma _{2}^{2},\rho \right)$ for the bivariate case, where $\sigma _{j}^{2}\sim \text {IG}\left( 0.01,0.01\right)$ for $j=1,2$ and $\rho \sim \text {U}\left( -1,1\right)$ to reflect our lack of information about these terms. The posterior computation involved in the IND prior is much less compared to the SS prior suggested in the Stan manual and can be done via a Gibbs sampler. Thus, throughout our simulation and real data analyses, the IND prior was used for this SS class for computational efficiency.

#### Scaled inverse Wishart prior

O’Malley and Zaslavsky^44^ developed a scaled inverse Wishart (SIW) prior that decomposes a covariance matrix differently such that $\varvec{\Sigma }=\Delta \mathbf {\textbf{Q}}\Delta$, where for the bivariate case, $\mathbf {\textbf{Q}}$ is an two dimensional unscaled matrix with the $\left( i,j\right)$ element $\mathbf {\textbf{Q}}_{ij}$ and $\Delta =\text {diag}\left( \delta _{1},\delta _{2}\right)$ . The SIW prior is defined as $\mathbf {\textbf{Q}}\sim \text {IW}\left( \nu ,\textbf{H}\right)$ and $\log \left( \delta _{j}\right) \overset{\text {ind}}{\sim }\text {N}\left( b_{j},\zeta _{j}^{2}\right)$, which implies that standard deviation $\sigma _{j}=\delta _{j}\sqrt{\mathbf {\mathbf {\textbf{Q}}}_{jj}}$ for $j=1,2$ and $\rho =\frac{\mathbf {\mathbf {\textbf{Q}}}_{12}}{\sqrt{\mathbf {\mathbf {\textbf{Q}}}_{11}\mathbf {\mathbf {\textbf{Q}}}_{22}}}$. Compared to the SS prior, the SIW prior avoids problematic transformation steps, yielding a more efficient sampling process. Following the specifications in Gelman and Hill^53^ and Akinc and Vandebroek^47^, we set $\textbf{H}=\textbf{I}$, $\nu =3$,$b_{j}=0$ and $\zeta _{j}^{2}=1$ for $j=1,2$.

## Simulation

We conducted two simulation studies focusing on meta-analysis of rare binary events: the first is to compare the performance of our Bayesian model under various covariance prior choices on the estimation of key model parameters in terms of bias, mean squared error (MSE) and coverage; the second is to assess the performance of our proposed IPQ method in comparison to existing GOF tests in terms of Type I error rates and statistical power. Our code is publicly available at https://github.com/chriszhangm/MetaGOF. In our numerical experiments, we used the default continuity correction factor of 0.5 for all frequentist methods unless otherwise stated. On the other hand, we did not adopt any continuity correction or eliminate studies containing zero events for Bayesian methods since they handle such studies automatically via incorporation of prior information into data analysis.

### Comparison of different covariance prior choices

We simulated data using the generalized REM in (2) to evaluate the performance of our Bayesian model with four covariance prior choices (i.e., IW, HIW, SIW and IND) in estimating (a) the overall treatment effect $\theta _{0}$, (b) the heterogeneity $\tau ^{2}$ and (c) the correlation coefficient $\rho$ , based on three metrics (bias, MSE and coverage). Specifically, for a parameter of interest (say $\kappa$, $\kappa$ can be $\theta _{0},\tau ^{2}$ or $\rho$), we define $\text {Bias}\left( \kappa \right) =\frac{\sum _{j=1}^{J}\left( \hat{\kappa }_{j}-\kappa \right) }{J}$ and $\text {MSE}\left( \kappa \right) =\frac{\sum _{j=1}^{J}\left( \hat{\kappa }_{j}-\kappa \right) ^{2}}{J}$, where $\hat{\kappa }_{j}$ is the corresponding estimate of $\kappa$ in the $j^{th}$ replicated dataset. We estimated $\theta _{0}$ and $\rho$ using the posterior mean, and $\tau ^{2}$ using the posterior median due to its heavily skewed distribution. The coverage probability was computed using $95\%$ equal-tail credible intervals.

To generate data from (2), we set $\mu _{1}=-5$, $\mu _{2}=-5+\theta _{0}$, and $\sigma _{1}^{2}=\sigma _{2}^{2}=0.5$. For (a), we varied $\theta _{0}\in \left\{ -1,-0.5,0,0.5,1\right\}$ and fixed $\rho =0$ so that $\tau ^{2}=1$ (recall that $\tau ^{2}=\sigma _{1}^{2}+\sigma _{2}^{2}-2\rho \sigma _{1}\sigma _{2}$) while for (b), we varied $\tau ^{2}\in \left\{ 0.2,0.4,...,1\right\}$ so that $\rho =1-\tau ^{2}$, and fixed $\theta _{0}=0$; for (c), we varied $\rho =\left\{ -0.8,-0.6,...,0.6,0.8\right\}$ so that $\tau ^{2}=1-\rho$, and fixed $\theta _{0}=0$. Then, for each setting, we simulated probabilities $p_{i1}=\frac{\exp \left( \phi _{i1}\right) }{1+\exp \left( \phi _{i1}\right) }$ and $p_{i2}=\frac{\exp \left( \phi _{i2}\right) }{1+\exp \left( \phi _{i2}\right) }$ for study $i=1,...,I.$ We considered three meta-analysis sizes $I=20,50,80$, and allowed different sample-size allocation ratios across studies by setting $n_{i2}=r_{i}n_{i1}$, where $\log _{2}r_{i}\sim \text {N}\left( 0,0.5\right)$. The number of subjects in the control group $n_{i1}$, was randomly drawn from 50 to 1000 for each study, and the number of events $x_{i1}$ or $x_{i2}$ was generated by $\text {Bin}\left( n_{i1},p_{i1}\right)$ or $\text {Bin}\left( n_{i2},p_{i2}\right)$.

**Figure 2:**
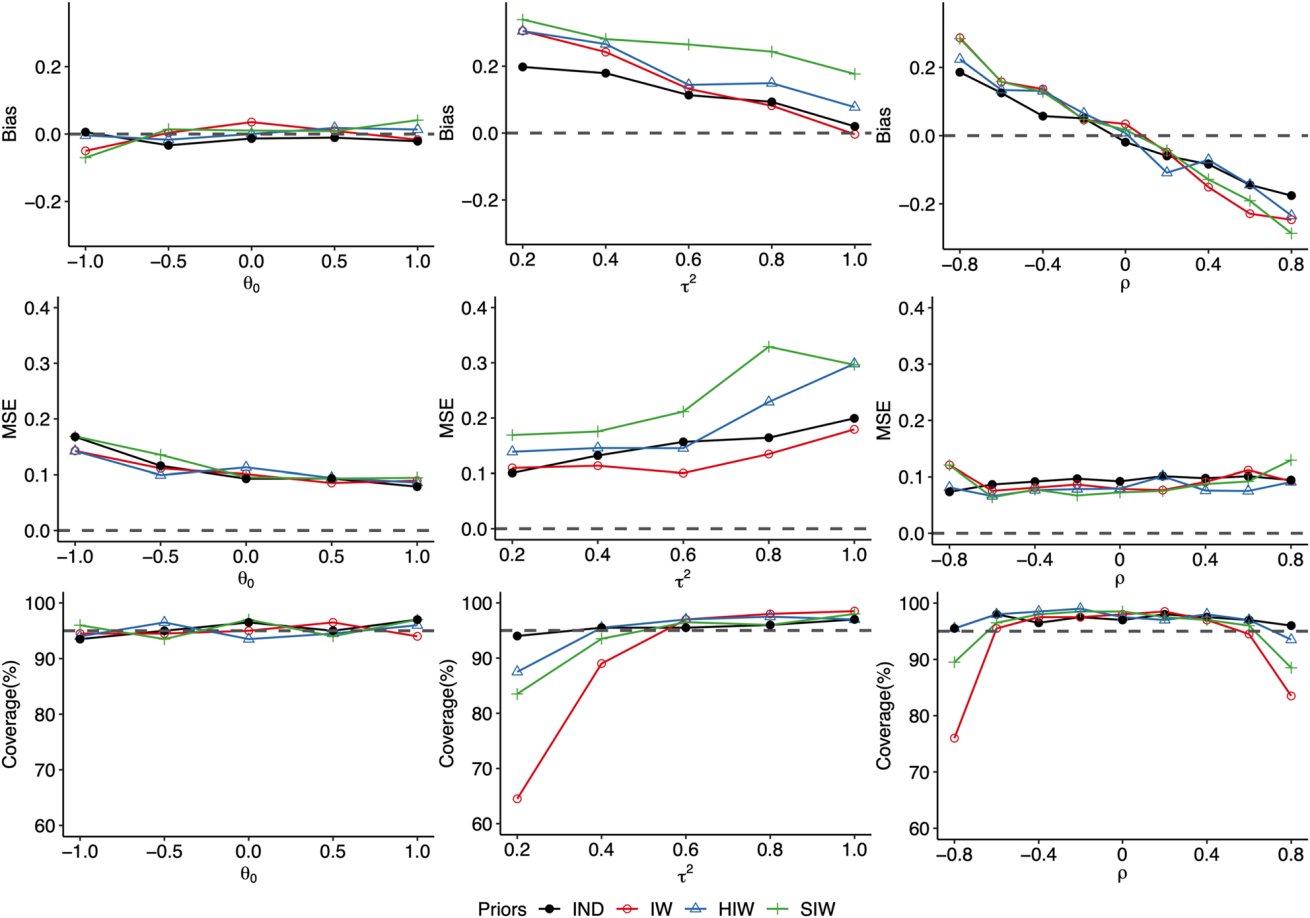
Comparison of bias, MSE and coverage results for Bayesian estimates of $\theta _{0}$, $\tau ^{2}$ and $\rho$ using the Inverse-Wishart prior (IW), the Hierarchical Inverse-Wishart prior (HIW), the Scaled Inverse-Wishart prior (SIW) and the independent prior (IND) for meta-analysis of rare binary events with $I=20$ studies. For each setting, results were generated using 200 replicates and the nominal coverage level was set as $95\%$.

In Figure 2, we report results based on 200 replicates for each setting with $I=20$, in which the three rows give bias, MSE and coverage results, and the three columns correspond to $\theta _{0}$, $\tau ^{2}$ and $\rho$, respectively. We observe that the performance of IPQ in estimating $\theta _{0}$ seems to be insensitive to the choices of different priors. For $\tau ^{2}$ and $\rho$, the IND prior generally outperforms other choices as it produces smaller bias and MSE as well as coverage closer to the nominal level 95% in most scenarios. Among the other three priors, although IW tends to do better in point estimation, it gives the worst coverage for both $\tau ^{2}$ and $\rho$ especially when $\tau ^{2}$ is small or $\left| \rho \right|$ is close to 1. This is not surprising, as mentioned before, the IW prior induces dependency between variance and correlation, which can bias the inference. For $I=50$ or 80 (results omitted here for brevity), while the discrepancies of the bias and coverage results using different priors become less salient, it is worth noting that IW still yields unsatisfactory coverage.

For GOF testing using the proposed IPQ method in this paper, the IND prior was used due to its demonstrated better performance and its simplicity.

### Performance evaluation of GOF testing

Our interest lies in conducting the GOF test to detect departures from a common assumption in meta-analysis that the true effect sizes $\theta _{i}$s of component studies are normally distributed. Using the generalized REM, for null cases, we generated $\phi _{i1}$ from normal distributions; for non-null cases, we generated $\phi _{i1}$ from one of four pre-specified non-normal distributions: (i) an exponential distribution with a rate of 1; (ii) a gamma distribution with a shape parameter of 4 and a scale parameter of 0.5, a unimodal right-skewed distribution; (iii) a mixture of two equal-weighted normal distributions: $\text {N}\left( 1,0.5\right)$ and $\text {N}\left( 4,0.5\right)$; (iv) a t distribution with number of degrees of freedom of 4. Then, with $z\sim \text {N}\left( 0,1\right)$, $\phi _{i2}$ given $\phi _{i1}$ was generated by $\phi _{i2}=\rho \frac{\sigma _{2}}{\sigma _{1}}\phi _{i1}+\sigma _{2}\sqrt{1-\rho ^{2}}z+\mu _{2}-\rho \frac{\sigma _{2}}{\sigma _{1}}\mu _{1}$ so that the correlation coefficient between $\left( \phi _{i1},\phi _{i2}\right)$ is $\rho$ and the mean and variance of $\phi _{ij}$ are $\mu _{j}$ and $\sigma _{j}^{2}$ for $j=1,2$, respectively. In other words, the conditional distribution of $\phi _{i2}$ given $\phi _{i1}$ is set to be normal in our simulation. Note that the four distributions of $\phi _{i1}$ cover different types of violation of the normality assumption, of which the first two cover skewness, the next covers multimodality, and the last covers heavy-tailedness. Generating the data in the above way would pass these types of violation to the distribution of $\theta _{i}$’s.

Without loss of generality, we set the means of $\phi _{i1}$ and $\phi _{i2}$ to be equal such that the overall effect $\theta _{0}=0$, $\mu =\mu _{1}=\mu _{2}\in \left\{ -5,-3,-2\right\}$, corresponding to $\left\{ 0.67\%,4.74\%,11.92\%\right\}$ in probability scale. We set $\sigma _{1}^{2}=0.5,\sigma _{2}^{2}=0.8$ for the null cases , and set $\sigma _{2}^{2}=0.8$ while $\sigma _{1}^{2}$ was determined by its distribution type specified above for the non-null cases(e.g., $\sigma _{1}^{2}=1$ for $\phi _{i1}$ from the exponential distribution). We further set the number of component studies $I\in \{20,50,80\}$, and $\rho \in \{-0.5,0,0.5\}$.

We compared our proposed method IPQ to six other approaches, including three frequentist-based approaches: the Naïve method that conducts the Shapiro-Wilk test on estimated effect sizes (log odds ratios) directly, the parametric bootstrap method (PB^8^) and the standardization method (STD^9^), and three Bayesian methods: the pivotal quantities method (PQ^10, 11^) using two cutoffs of 0.25 and 0.1, the posterior predictive check (PPC^15^) using the discrepancy function recommended in Sinharay and Stern^54^, defined as $T\left( \varvec{\theta }\right) =\left| \max \left( \varvec{\theta }\right) -\text {median}\left( \varvec{\theta }\right) \right| -\left| \min \left( \varvec{\theta }\right) -\text {median}\left( \mathbf {\varvec{\theta }}\right) \right|$ with $\varvec{\theta }=(\theta _{i})_{i=1}^{I}$, and the sampled posterior check (SPC^21, 22^) using the same discrepancy function. We set the significance level $\alpha =0.05$. For all frequentist approaches (PB, STD and Naive) and the proposed IPQ, we reject the normality assumption if the *p* value is less than 0.05. For PPC or SPC, we reject the null when the posterior predictive *p* value (PPP) is below 0.025 or above 0.975^22^; for PQ, if the minimum *p* value upper bound $p_{\text {min}}$ is less than the chosen cutoff (0.25 or 0.1), we reject the null. As mentioned earlier, for either PPC or PQ, the reference distribution of PPP or $p_{\text {min}}$ is not uniform(0,1) even in an asymptotic sense, and so we do not expect that they maintain the Type I error rate. However, the cutoff value 0.1 for $p_{\text {min}}$ in the PQ method was chosen via preliminary simulation because it can offer error rates much closer to 0.05 in most of the simulation scenarios, compared to the rule-of-thumb value of 0.25. We simulated 200 replicates for each setting, and reported Type I error rates for data from null cases and statistical power otherwise.

**Figure 3:**
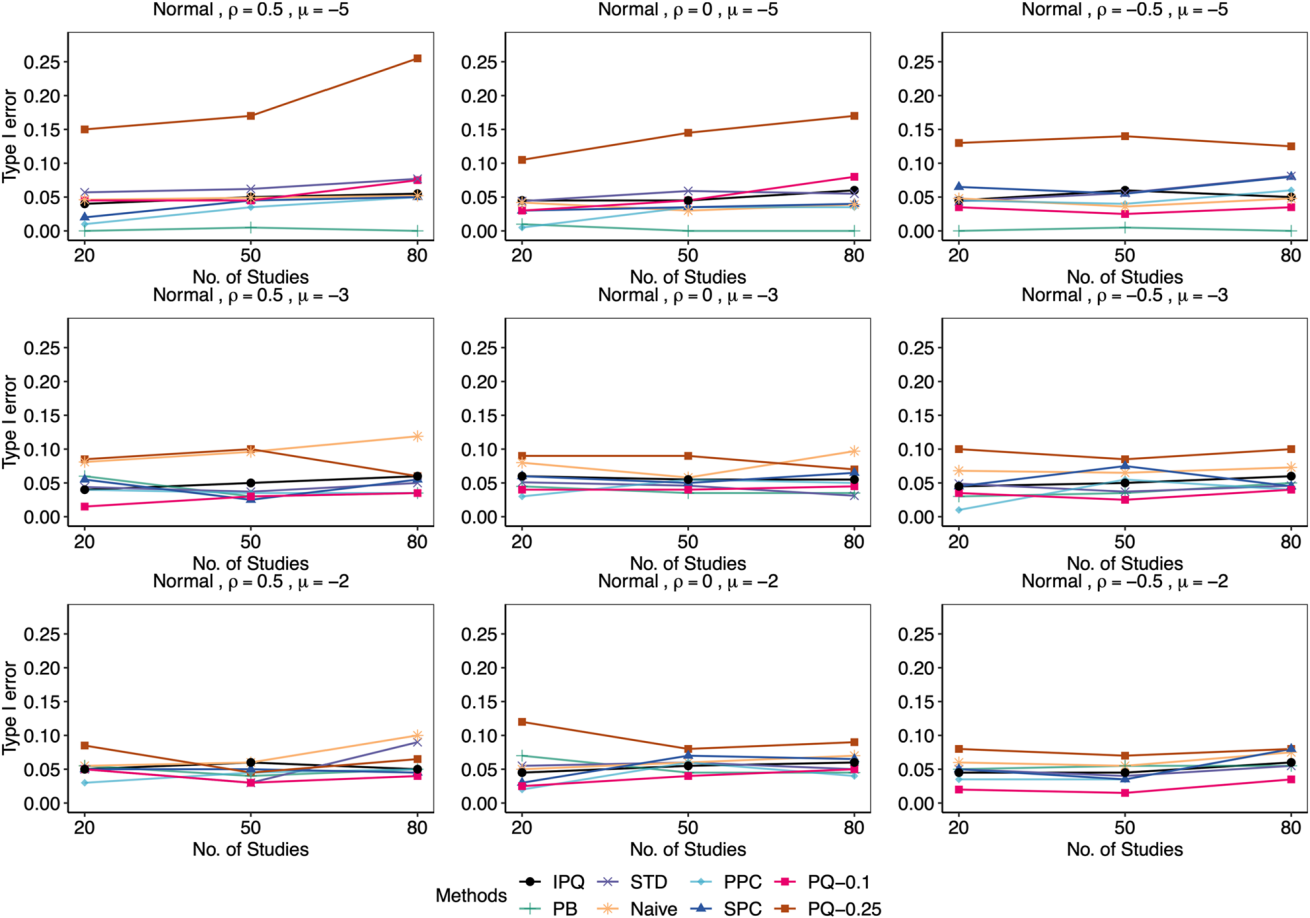
Comparison of empirical Type I error rates by proposed IPQ, parametric bootstrap method (PB), standardization method (STD), Naïve method (Naive), Posterior Predictive Check (PPC), Sampled Posterior Check (SPC), and Pivotal Quantities method with cutoffs of 0.1 and 0.25 (PQ-0.1, PQ-0.25). Data were generated from the null cases with different $(I,\mu ,\rho )$ combinations, each 200 replicates. Tests were conducted at the significant level $\alpha =0.05$.

Figure 3 reports the Type I error rates for all methods in various settings. The IPQ, STD, and SPC methods demonstrate superior performance, as they maintain an error rate close to the nominal value of 0.05 regardless of $\left\{ I,\mu ,\rho \right\}$. Conversely, PQ-0.25 (the PQ method with the recommended cutoff of 0.25^11^) frequently produces severely inflated Type I error rates, particularly as the event of interest becomes rarer, while PQ-0.1 performs much better in general. Therefore, we exclude PQ-0.25 from our power results below. Among the remaining three methods, PPC and PB are often conservative for rarer events (i.e., $\mu =-5$), exhibiting Type I error rates below 0.05, while Naive tends to have inflated rates for less rare events.

**Figure 4:**
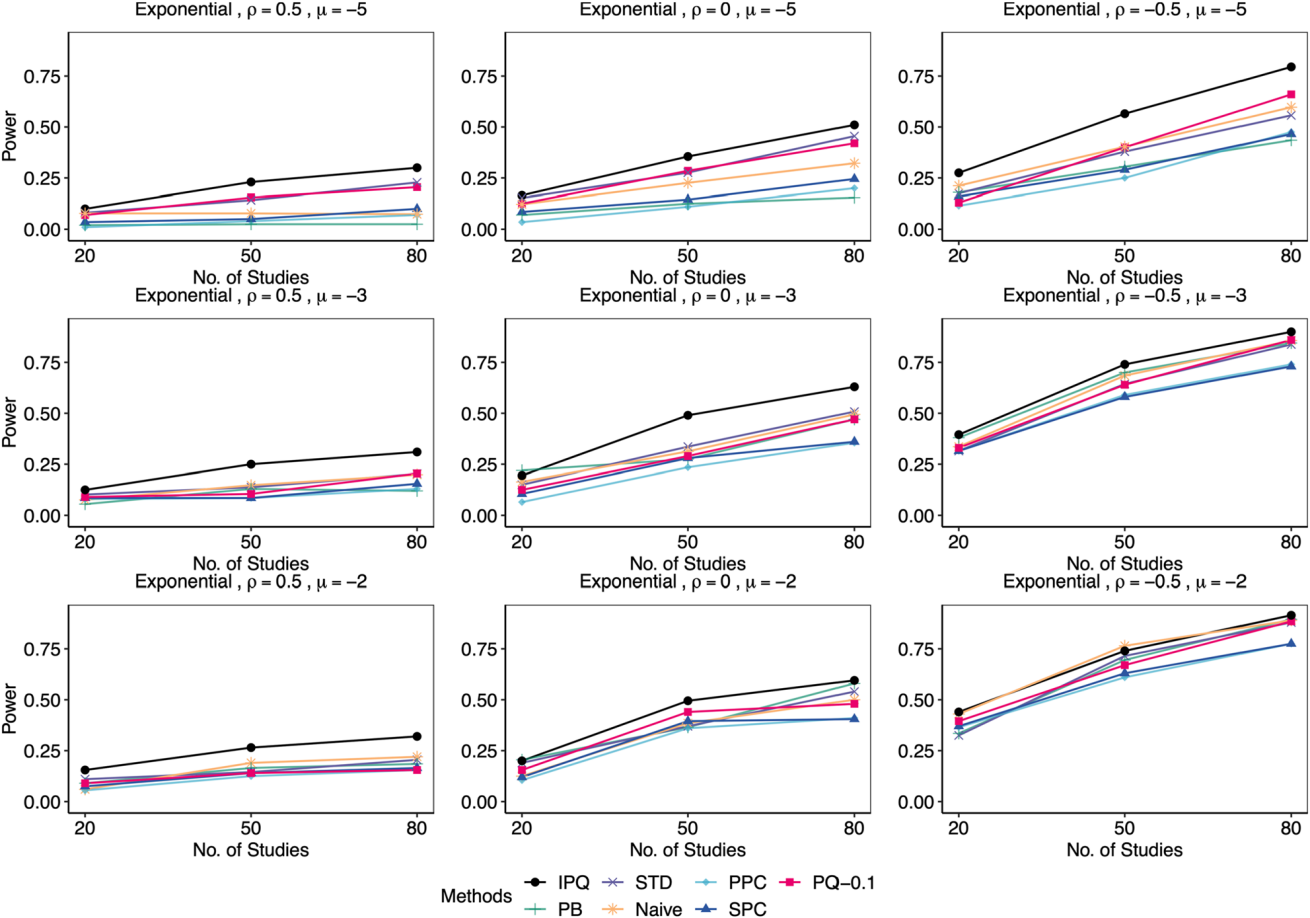
Comparison of empirical power by proposed IPQ, parametric bootstrap method (PB), standardization method (STD), Naïve method (Naive), Posterior Predictive Check (PPC), Sampled Posterior Check (SPC), and Pivotal Quantities method with a cutoff of 0.1(PQ-0.1). Data were generated from the non-null cases, where $\phi _{i1}\sim \text {Exp}(1)$, with different $(I,\mu ,\rho )$ combinations, each 200 replicates. Tests were conducted at the significant level $\alpha =0.05$.

**Figure 5:**
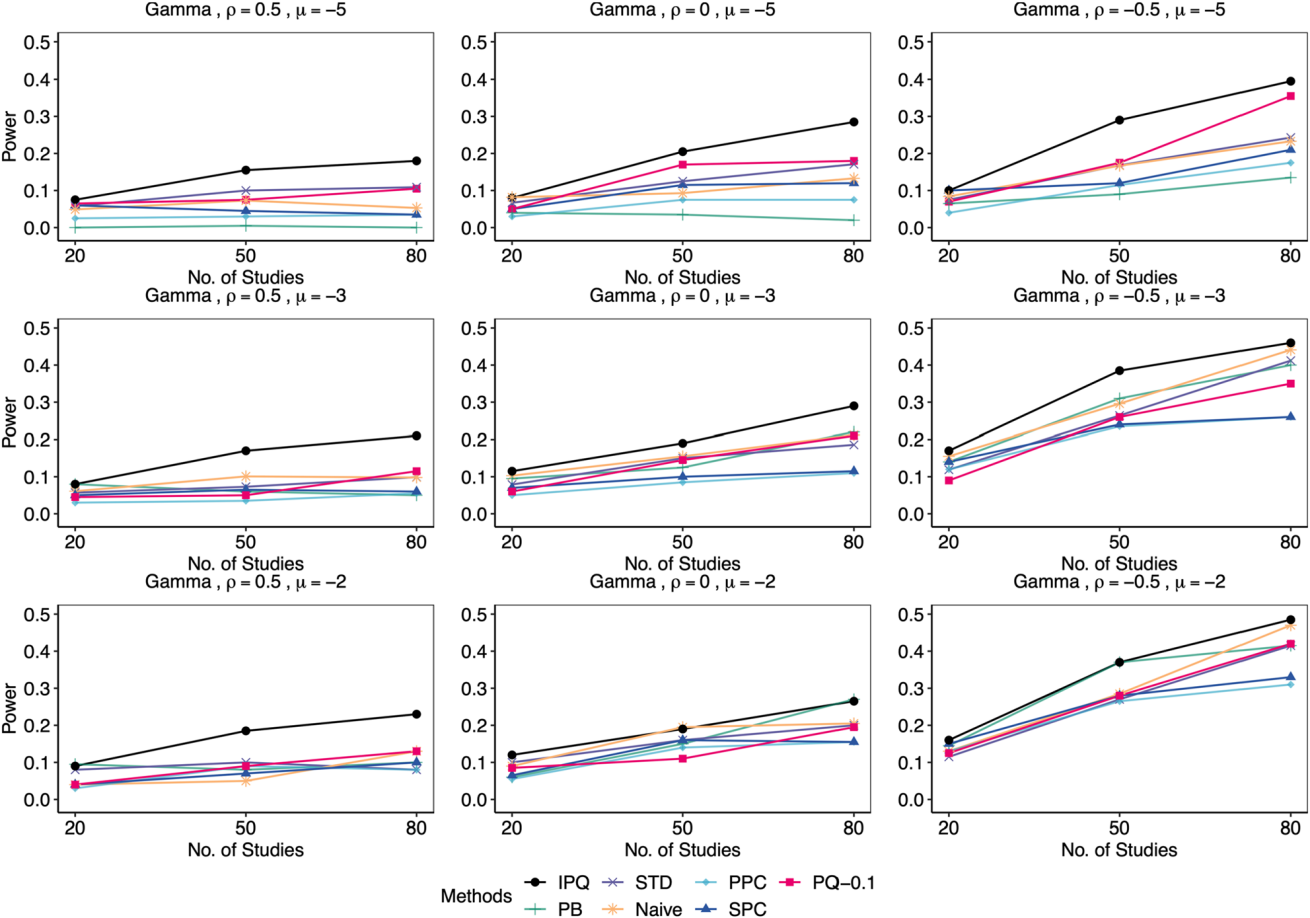
Comparison of empirical power by proposed IPQ, parametric bootstrap method (PB), standardization method (STD), Naïve method (Naive), Posterior Predictive Check (PPC), Sampled Posterior Check (SPC), and Pivotal Quantities method with cutoff 0.1(PQ-0.1). Here, data were generated from the non-null cases, where $\phi _{i1}\sim \text {Gamma}(4,0.5)$, with different $(I,\mu ,\rho )$ combinations, each 200 replicates. Tests were conducted at the significant level $\alpha =0.05$.

**Figure 6:**
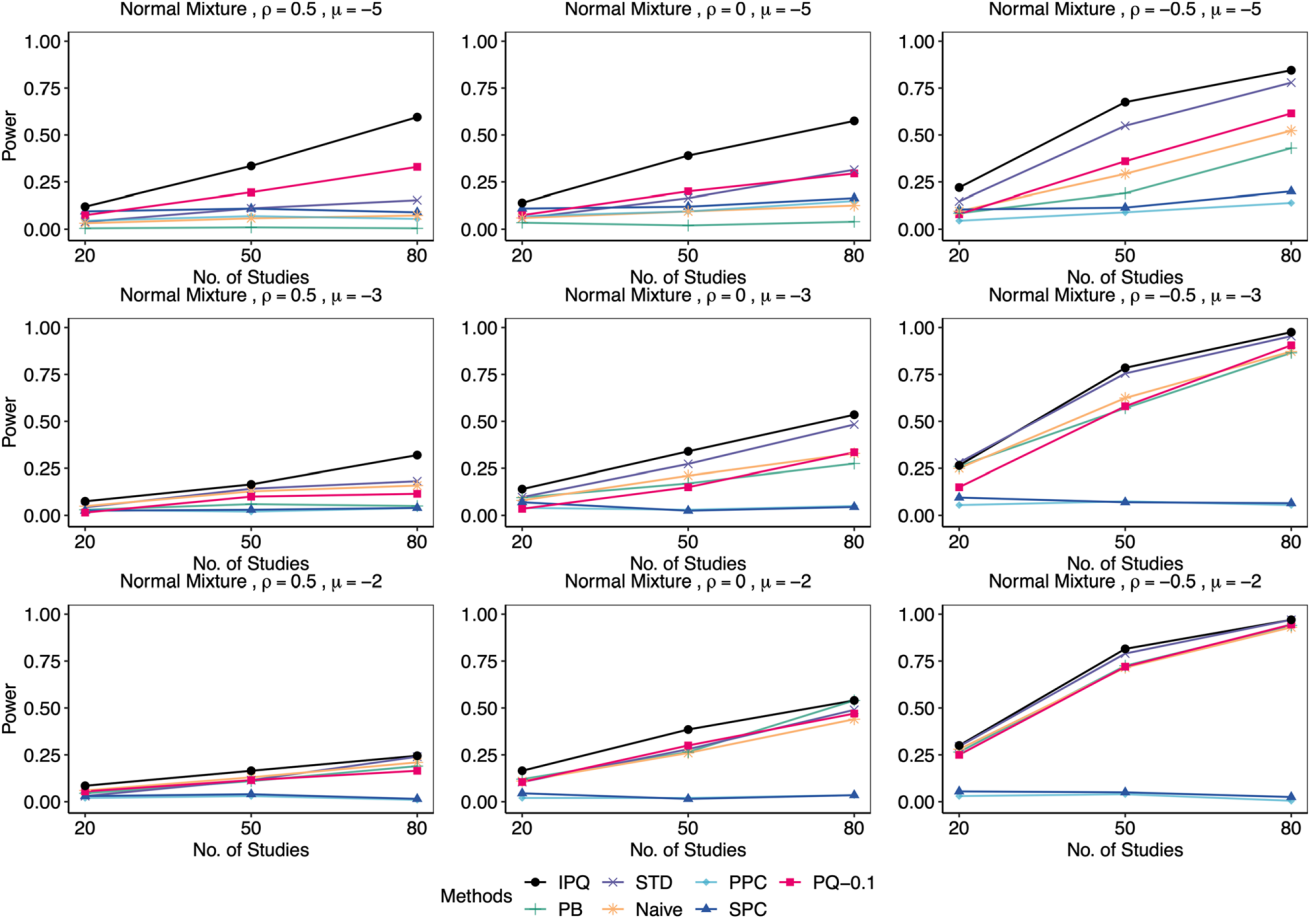
Comparison of empirical power by proposed IPQ, parametric bootstrap method (PB), standardization method (STD), Naïve method (Naive), Posterior Predictive Check (PPC), Sampled Posterior Check (SPC), and Pivotal Quantities method with a cutoff of 0.1(PQ-0.1). Here, data were generated from the non-null cases, where $\phi _{i1}\sim 0.5\text {N}(1,0.5)+0.5\text {N}(4,0.5)$, with different $(I,\mu ,\rho )$ combinations, each 200 replicates. Tests were conducted at the significant level $\alpha =0.05$.

**Figure 7:**
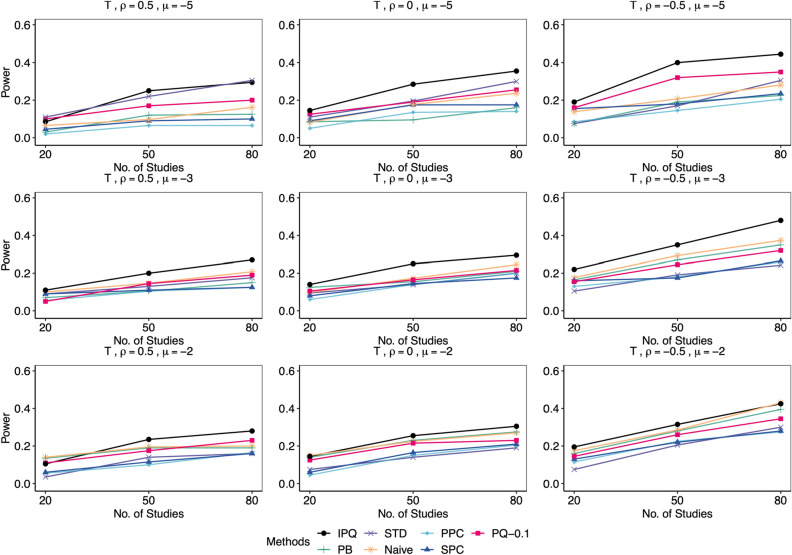
Comparison of empirical power by proposed IPQ, parametric bootstrap method (PB), standardization method (STD), Naïve method (Naive), Posterior Predictive Check (PPC), Sampled Posterior Check (SPC), and Pivotal Quantities method with a cutoff of 0.1(PQ-0.1). Here, data were generated from the non-null cases, where $\phi _{i1}\sim \text {t}_{4}$, with different $(I,\mu ,\rho )$ combinations, each 200 replicates. Tests were conducted at the significant level $\alpha =0.05$.

Figures 4, 5, 6, 7 display power results with different underlying distributions of $\phi _{i1}$. We observe that all approaches tend to report higher power as *I* increases or $\rho$ decreases. Also, the differences in power among the methods become smaller as $\mu$ goes up. This is perhaps because some methods in the bottom group such as PB improve significantly while the proposed IPQ, as the best overall method, appears to be much less sensitive to the change of $\mu$. Figures 4 and 5 present power results for skewed distributions (i.e., exponential and gamma distributions). IPQ is the best in nearly all scenarios, followed by PQ-0.1 and STD. Naive often stands somewhere in the middle among all. PB tends to perform poorly, except for larger $\mu$ and smaller $\rho$. SPC reports slightly better results than PPC, while both methods provide the worst overall results, particularly with large $\rho$. Figure 6 presents outcomes for a multimodal distribution (i.e., normal mixture). IPQ is a clear winner and provides the highest power in all settings. Among the others, STD and PQ-0.1 usually perform better, followed by Naïve and PB. SPC and PPC consistently give the worst results that show almost no power. Figure 7 displays power results for $t_{4}$, a symmetric and heavy-tailed distribution, where again, IPQ outperforms other methods while PPC and SPC tend to perform the worst.

To summarize, the proposed IPQ maintains the Type I error rate at the target level well and offers the highest statistical power for various departures from the normality assumption compared to alternative approaches.

## Data examples

We applied our IPQ method, along with six other methods (PB, STD, Naïve, PPC, SPC and PQ-0.1) to three real data sets of meta-analysis, for testing the normality assumption about the distribution of true effect sizes across component studies. The first involves hand-eye dominance data, the second involves diabetes data, and the third involves lung cancer data.

**Figure 8:**
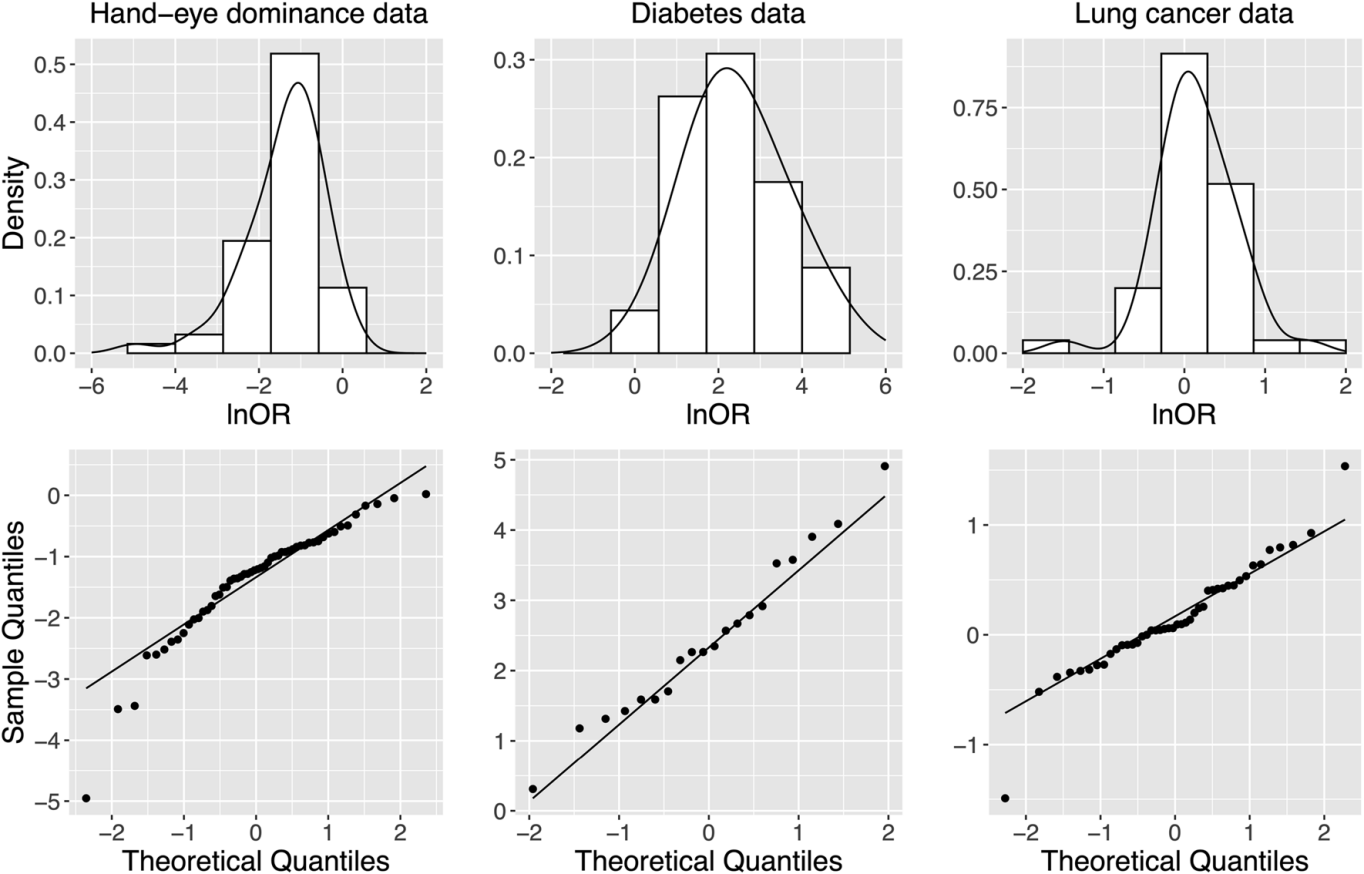
The histogram and density plots (top) and Quantile-Quantile plot (bottom) of the observed effect sizes measured by log odds ratio (lnOR). The left panel is for hand-eye dominance data, the middle panel is for diabetes data and the right panel is for lung cancer data.

Bourassa^55^ conducted a meta-analysis of 54 studies to investigate the hand-eye dominance association (see Table A1 for detailed data in Supplementary Material). The study found that the hand-eye concordance was larger than one, indicating left-handed people tended to have left-eyed dominance, and the same was true for right-handed people. The meta-analysis included 54,087 subjects, summarized in $2\times 2$ tables of four categories: left-handed/left-eyed, left-handed/right-eyed, right-handed/left-eyed and right-handed/right-eyed. We considered the event of interest to be “ left-handed,” with the control and case groups being “ left-eyed” and “ right-eyed,” respectively. The overall incident rates for the control and case groups are about $6\%$ and $18.5\%$, which are $-2.75$ and $-1.48$ on a logit scale equivalently. The left panel of Figure 8 displays the histogram, density and quantile-quantile plots of the observed log odds ratio, revealing a left-skewed distribution.

Bellamy et al.^56^ conducted a meta-analysis of 20 studies to investigate the association between Type 2 diabetes mellitus and gestational diabetes (see Table A2 for detailed data in Supplementary Material). The analysis revealed that women with gestational diabetes had an increased risk of developing type 2 diabetes. The study included 675,455 subjects, of which 31,867 had Type 2 diabetes. Among the control groups (no gestational diabetes), 6,862 subjects had Type 2 diabetes, indicating an overall incident rate of ~$1.1\%$ (or $-4.53$ on a logit scale). For the case groups (with gestational diabetes), 3997 of them had Type 2 diabetes, resulting in an incident rate of ~$12.5\%$ (or $-1.94$ on a logit scale). The middle panel of Figure 8 shows the histogram, density, and quantile-quantile plots of the observed log odds ratio, suggesting a unimodal, symmetric, and bell-shaped curve.

Feng et al.^57^ conducted a meta-analysis of 44 studies to evaluate the association between GSTP1 gene polymorphism and the risk of lung cancer (see Table A3 for detailed data in Supplementary Material). The event of interest is considered the GG genotype of GSTP1. The study included 26,516 subjects, of which 2763 had the GG genotype. Among the control (no lung cancer) and case (lung cancer) groups, 1406 and 1357 subjects had the GG genotype, implying overall incident rates of 10.0 (or $-2.19$ on a logit scale) and 10.8 (or $-2.11$ on a logit scale), respectively. The right panel of Figure 8 reveals the histogram, density, and quantile-quantile plots of the observed log odds ratio, showing a roughly symmetric curve but with heavy tails on both sides.

Table 1 shows that for the hand-eye dominance data, at the significance level $\alpha =0.05$, all methods except for STD reject the null hypothesis, indicating a departure from the assumed normality. Note that among the existing methods, STD was quite competitive. Nevertheless, it failed in this specific example. On the other hand, PPC and SPC tend to be conservative in rejecting the null but worked here. For the diabetes data, Table 1 shows that all methods have the same conclusion: there is no evidence against the normality.

For the GSTP1 gene polymorphism and lung cancer data, Table 1 shows that Naïve, PQ-0.1 and IPQ reject the null hypothesis while other methods do not provide evidence against normality. As shown in Figure 7, given the symmetric and heavy-tailed distribution, IPQ offered the highest power across all the cases. When $\mu =-2$, similar to the overall incidence rates in this dataset, Naïve and PQ-0.1 performed relatively well. However, STD, PPC, and SPC performed poorly under the cases of $\mu =-2$. Therefore, we recommend avoiding the normality assumption in this example.

In summary, IPQ performs consistently well across all three real data examples, while PB, STD, PPC, and SPC sometimes fail. Although Naïve and PQ-0.1 also demonstrate good performance here, they are less satisfactory in our simulation studies. As such, IPQ is the recommended method of choice in this context.

**Table 1:** *P* values of the GOF tests for three meta-analyses involving (i) hand-eye dominance data, (ii) type 2 diabetes mellitus and gestational diabetes data, and (iii) GSTP1 gene polymorphism and lung cancer data. Note that for PPC or SPC, the posterior predictive *p* value is reported and for PQ-0.1, the minimum *p* value upper bound $p_{\text {min}}$ is reported.

Methods	Hand-eye dominance	Diabetes	Lung cancer
PB	0.021	0.932	0.753
STD	0.069	0.835	0.460
Naïve	<0.001	0.921	0.034
PPC	0.999	0.277	0.666
SPC	0.998	0.245	0.714
PQ-0.1	0.031	0.707	0.022
IPQ	0.007	0.985	0.021

## Discussion

Meta-analysis commonly assumes that actual effect sizes from component studies follow a normal distribution for mathematical convenience, despite a lack of formal justification for this assumption. In practice, however, this assumption can be frequently violated, potentially leading to inaccurate conclusions. To address this issue, we propose a novel goodness-of-fit (GOF) test called Improved Pivotal Quantities (IPQ) for testing this assumption in the context of meta-analysis of rare binary outcomes, where the effect size is measured by log odds ratio.

The proposed IPQ method builds upon the strengths of the original PQ approach^10^, which is conceptually simple and efficient in detecting model misfit at any level of a hierarchical model without additional computational costs. However, the original PQ method employs the probability bound as a criterion to determine model misfit, which can result in inflated Type I error rates when used with the rule-of-thumb cutoff of 0.25. This highlights the need for selecting new cutoff values that are tailored to different applications. To address this limitation, our IPQ method improves the decision-making process of PQ by adopting the Cauchy combination idea^12^ to account for dependent *p* value. In addition, given sparse data such as tables with zero events, IPQ naturally incorporates all data, without requiring artificial corrections due to its Bayesian model formulation.

In fact, IPQ is a hybrid approach. It adopts the frequentist framework for hypothesis testing, since it uses the Cauchy combination test to obtain a *p* value, from which the final conclusion is drawn. On the other hand, it constructs the test statistics by incorporating a Bayesian idea through Markov Chain Monte Carlo methods. We further note that, because of the use of pivotal quantities, the sampling distribution of the proposed test statistics, evaluated at posterior samples, is known and invariant (i.e., N(0,1)) under the null hypothesis. The set of posterior draws used to construct the test statistics is from the same data (i.e., the true observed data rather than any “fake” data).

Simulation results indicate that IPQ maintains well-controlled Type I error rates while achieving higher statistical power than other approaches in most scenarios. To demonstrate the effectiveness of our method, we provide examples of three real datasets. Specifically, our results suggest that the normality assumption should be avoided for the hand-eye dominance dataset^55^ and the GSTP1 gene polymorphism and lung cancer dataset^57^, while it is likely to hold for the diabetes dataset^56^. In situations where the normality assumption does not hold, it becomes imperative to explore alternative distributions, such as those characterized by heavy tails (e.g., t distributions) or skewness (e.g., gamma distributions), in order to more accurately capture the characteristics of observed data. Alternatively, one can employ nonparametric methods for estimating treatment effects^58^ and for estimating heterogeneity^59–61^. Furthermore, in scenarios where a meta-analysis involves a small number of studies, a situation commonly encountered in practice, alternative frameworks such as Bayesian model averaging may yield more reliable outcomes.

Although our focus is primarily on rare binary events, the IPQ method is directly applicable to meta-analysis of any binary data. However, we believe that the gain in performance for common binary events may not be as significant as that for rare binary events. As demonstrated in our simulation studies, the differences in power between our method and other approaches diminish when increasing the background incidence rate. Moreover, IPQ can be extended beyond testing normality to other scenarios where an appropriate test statistic can be designed to measure the discrepancy. In conclusion, our IPQ method is useful for detecting model misfits and selecting appropriate statistical models for different applications, particularly in scenarios where sparse data are present or when the normality assumption is in question.

### Supplementary Information

Supplementary Information.

## Data Availability

The data that support the findings of this study are included in Supplementary Material.
