# Supplementary material for: Goodness-of-fit testing for meta-analysis of rare binary events
Source: Sci Rep. 2023 Oct 18;13:17712. doi: 10.1038/s41598-023-44638-x (PMC10584850; doi:10.1038/s41598-023-44638-x)
Supplement: Supplementary file 1 — Supplementary Information. [file 41598_2023_44638_MOESM1_ESM.pdf]

Supplementary Material
"A Bayesian Goodness-of-Fit Test for Meta-Analysis of Rare Binary Events"

By Ming Zhang, Olivia Y. Xiao, Johan Lim, Xinlei Wang

This supplementary document includes data tables that are mentioned in the paper.

A1 Handedness and eye-dominance data

	Case (Right-eye)		Control (Left-eye)			Case (Right-eye)		Control (Left-eye)	
Study	N	Event #	N	Event #	Study	N	Event #	N	Event #
1	223	93	777	17	28	67	11	133	11
2	445	140	788	91	29	136	18	175	20
3	59	16	128	14	30	77	38	48	14
4	699	102	1995	97	31	35	19	39	6
5	55	17	94	14	32	191	19	364	12
6	62	10	172	2	33	1486	411	3661	198
7	22	7	171	2	34	60	9	131	8
8	19	4	40	2	35	68	26	124	34
9	26	2	42	3	36	47	4	67	2
10	21	3	43	4	37	49	18	94	19
11	33	4	92	3	38	302	31	551	8
12	22	2	50	1	39	191	11	374	21
13	22	3	47	2	40	227	37	287	27
14	128	27	261	0	41	2968	467	3764	300
15	68	9	109	11	42	84	16	348	15
16	28	5	40	2	43	42	10	86	2
17	157	20	340	13	44	3266	563	7247	320
18	20	6	42	8	45	19	5	38	1
19	39	8	61	5	46	48	19	138	6
20	1828	241	3651	211	47	94	46	203	32
21	46	19	43	10	48	232	89	454	70
22	35	2	86	2	49	41	25	121	53
23	37	8	58	2	50	515	141	1573	54
24	30	13	57	15	51	112	23	522	17
25	107	10	206	20	52	160	32	453	13
26	2957	429	4729	311	53	183	20	388	12
27	22	10	58	10	54	159	30	455	8

Table A1: Handedness and Eye-dominance data in Bourassa (1996). N: the number of patients, Event: left-handed.

A2 Type 2 diabetes mellitus and gestational diabetes data

	Case(GDM)		Control (no GDM)	
Study	N	Event #	N	Event #
1	21823	2874	6628	637341
2	71	620	22	868
3	21	68	0	39
4	43	166	150	2242
5	53	295	1	111
6	405	5470	16	783
7	6	70	7	108
8	13	35	8	489
9	7	23	0	11
10	44	696	0	435
11	21	229	0	70
12	10	28	1	61
13	15	45	0	52
14	105	801	1	39
15	10	28	7	431
16	15	45	0	35
17	33	241	0	57
18	14	47	3	47
19	224	615	18	328
20	5	145	0	41

Table A2: Type 2 diabetes mellitus and gestational diabetes data in Bellamy et al. (2009). N: the number of patients, Event: Type 2 diabetes, GDM: gestational diabetes mellitus.

A3 GSTP1 gene and lung cancer data

	Case (Cancer)		Control (no cancer)			Case (Cancer)		Control (no cancer)	
Study	N	Event #	N	Event #	Study	N	Event #	N	Event #
1	138	22	297	27	23	29	6	29	4
2	178	26	199	18	24	1095	110	626	84
3	150	17	172	22	25	1921	220	1343	141
4	169	9	241	14	26	249	116	260	115
5	47	0	122	5	27	429	55	766	94
6	358	17	257	8	28	211	21	211	10
7	164	17	200	20	29	317	16	353	12
8	388	38	353	35	30	213	11	213	7
9	93	6	151	13	31	200	25	264	30
10	85	15	163	14	32	151	5	151	6
11	251	30	264	20	33	319	9	381	2
12	282	29	541	54	34	93	3	253	15
13	112	1	119	1	35	617	69	1257	136
14	362	35	419	44	36	89	5	108	9
15	229	71	197	65	37	462	19	379	6
16	446	62	622	70	38	100	37	125	12
17	235	31	233	39	39	118	13	290	22
18	12	0	23	0	40	788	97	788	92
19	228	22	288	38	41	142	23	190	26
20	89	13	119	19	42	198	33	233	27
21	227	4	227	3	43	170	5	270	5
22	112	15	151	18	44	150	9	152	4

Table A3: GSTP1 gene and lung cancer in Feng et al. (2012). N: the number of patients, Event: the GG genotype of GSTP1 gene.

References

Bellamy, Leanne et al. (May 2009). "Type 2 diabetes mellitus after gestational diabetes: a systematic review and meta-analysis". In: *The Lancet* 373.9677, pp. 1773–1779.

Bourassa, D.C. (Mar. 1996). "Handedness and Eye-dominance: A Meta-analysis of Their Relationship". In: *Laterality* 1.1, pp. 5–34.

Feng, Xu et al. (2012). "Association of glutathione S-transferase P1 gene polymorphism with the susceptibility of lung cancer". In: *Molecular biology reports* 39, pp. 10313–10323.
